# Pockels laser directly driving ultrafast optical metrology

**DOI:** 10.1038/s41377-025-01872-4

**Published:** 2025-05-30

**Authors:** Shixin Xue, Mingxiao Li, Raymond Lopez-rios, Jingwei Ling, Zhengdong Gao, Qili Hu, Tian Qiu, Jeremy Staffa, Lin Chang, Heming Wang, Chao Xiang, John E. Bowers, Qiang Lin

**Affiliations:** 1 Department of Electrical and Computer Engineering, University of Rochester, Rochester, NY USA; 2 Department of Electrical and Computer Engineering, University of California Santa Barbara, Santa Barbara, CA USA; 3 Institute of Optics, University of Rochester, Rochester, NY USA

**Keywords:** Integrated optics, Semiconductor lasers

## Abstract

The invention of the laser unleashed the potential of optical metrology, leading to numerous advancements in modern science and technology. This reliance on lasers, however, also introduces a bottleneck for precision optical metrology, as it requires sophisticated photonic infrastructure for precise laser-wave control, leading to limited metrology performance and significant system complexity. Here, we take a key step toward overcoming this challenge by demonstrating a Pockels laser with multifunctional capabilities that elevate optical metrology to a new level. The chip-scale laser achieves a narrow intrinsic linewidth down to 167 Hz and a broad mode-hop-free tuning range up to 24 GHz. In particular, it delivers an unprecedented frequency chirping rate of up to 20 EHz/s and an exceptional modulation bandwidth exceeding 10 GHz, both of which are orders of magnitude greater than those of existing lasers. Leveraging this laser, we successfully achieve velocimetry at 40 m/s over a short distance of 0.4 m, and measurable velocities up to the first cosmic velocity at 1 m away—a feat unattainable with conventional ranging approaches. At the same time, we achieve distance metrology with a ranging resolution of <2 cm. Furthermore, for the first time to our knowledge, we implement a dramatically simplified architecture for laser frequency stabilization by directly locking the laser to an external reference gas cell without requiring additional external light control. This approach enables long-term laser stability with a frequency fluctuation of only ±6.5 MHz over 60 min. The demonstrated Pockels laser combines elegantly high laser coherence with ultrafast frequency reconfigurability and superior multifunctional capability. We envision its profound impact across diverse fields including communication, sensing, autonomous driving, quantum information processing, and beyond.

## Introduction

Optical metrology has emerged as one of the most effective ways for humans to observe the world. Using light as a probe offers unprecedented advantages in measuring objects and physical quantities. In fundamental science, precision optical metrology plays an indispensable role in the most delicate experiments such as optical clock, gravitational wave detection, and dark-matter observation^1–3^. In daily life, the noncontact nature of optical measurement enables the capture of the physical information of the target in a fast and precise way, which benefits diverse applications ranging from self-driving, robotics, to advanced manufacturing^4–7^.

**Fig. 1:**
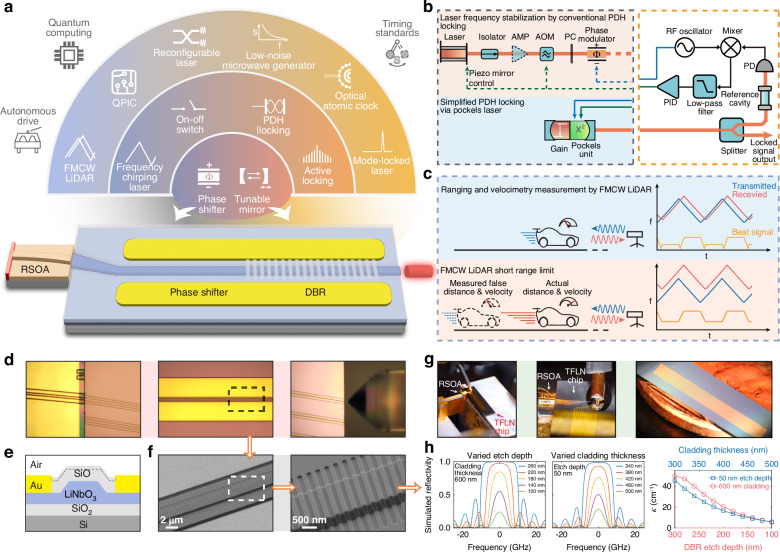
Concept of integrated Pockels laser for optical metrology applications. **a** Outlook of Pockels lasers and their potential applications. Bottom: Schematic of the integrated eDBR-based Pockels laser. **b** Schematic of laser frequency stabilization via PDH locking. Top: Conventional PDH locking configuration. Bottom: Simplified PDH locking using Pockels laser. AOM acousto-optic modulator, PC polarization controller, AMP optical amplifier, PD photodetector. **c** Schematic of distance metrology and velocimetry measurements by FMCW LiDAR. Top: normal detection regime for FMCW LiDAR. Bottom: false detection caused by large velocity and/or short distance. **d** Optical microscopic images of the integrated eDBR-based Pockels laser. **e** Schematic of the cross-section of the DBR section. **f** Scanning electron microscopic images of the DBR section. **g** Left and middle: Photo and its zoomed-in view of the laser device under testing. Right: Photo of the full TFLN external cavity PIC chip (compared with a penny coin shown in the background). **h** Simulated reflection spectrum (left two figures) and coupling coefficient *κ* (right figure) of the DBR at varied etch depth and cladding thickness. The DBR grating in simulation has a period of 400 nm (corresponding to an optical frequency centered around 192.8 THz), with a total number of 10,000 periods

The essential element lying in the heart of optical metrology is the generation and control of coherent laser waves. Although significant advances have been made in recent years in the generation of high-coherence lasers, particularly in narrow-linewidth semiconductor lasers^8–12^, controlling laser waves for metrologic purposes remains fairly complicated. The realization of a complete metrologic functionality generally requires sophisticated photonic infrastructures external to the laser for manipulating the frequency, phase, power, polarization, and/or optical path of the laser wave. This complexity leads to the bulky nature of current metrological systems, which not only hinders system miniaturization but also limits the metrology performance. Taking the optical clock^1^ as an example, lasers are required to stably lock to certain atomic transition lines and/or to a reference cavity for proper operation. This is generally realized via the Pound–Drever–Hall (PDH) technique^13,14^ that requires electro-optic components for frequency modulation, acoustic-optic devices for beam deflection, and piezoelectric (PZE) means for delicate servo control of the laser cavity^15,16^, all of which can only be done external to the laser diode (Fig. 1b). The same complexity is inherited in an atomic quantum sensor^17,18^ or an optical quantum computing system^19,20^ whose operation also relies on precise laser accessing of atomic transitions.

On the other hand, in distance metrology and velocimetry, the frequency-modulated continuous-wave (FMCW) light detection and ranging (LiDAR) technique has attracted significant interest recently, given its simultaneous ranging and velocimetry capabilities and its superior environmental immunity. It is considered a crucial metrology technique for supporting fully self-driving in future motor vehicles^4,5^. However, the dynamic range of ranging and velocimetry in FMCW LiDAR essentially depends on the speed and linearity of laser frequency chirping, which is significantly beyond the reach of current existing lasers. Consequently, FMCW LiDAR has to rely on complicated modulation and optoelectronic feedback control external to the laser to improve frequency chirping^21–23^, whose limited performance seriously impacts the capability of velocity detection and could lead to severe false judgment in self-driving motor vehicles (Fig. 1c).

In this work, we take a key step toward resolving these challenges by demonstrating a new type of laser that can directly drive optical metrology systems in a dramatically simplified configuration while achieving significantly enhanced performance. By utilizing our recently developed Pockels laser integration strategy and a novel co-tuned phase-distributed Bragg reflector (DBR) structure, we successfully combine a narrow-linewidth laser with unprecedentedly fast-speed, wide-range frequency reconfigurability. The chip-scale integrated laser exhibits a narrow intrinsic linewidth down to 167 Hz, a broad mode-hop-free (MHF) tuning range of 24 GHz, and a record-high frequency chirping rate of up to 2 × 10^19^ Hz/s. Moreover, the laser offers an enormous modulation bandwidth of up to >10 GHz for direct feedback locking inside the laser cavity, orders of magnitude larger than any existing lasers.

The superior performance of the demonstrated laser now opens up a significant opportunity to significantly advance a variety of optical metrology applications. To showcase this capability, we use the laser to implement an FMCW LiDAR system and achieve ultrafast ranging and velocimetry of distant objects, with a measured velocity of up to 40 m/s at a very short distance of 0.4 m that is inaccessible to current state-of-the-art FMCW LiDAR systems. At the same time, we also achieve two-dimensional imaging with a ranging resolution of <2.0 cm. On the other hand, we successfully demonstrate a dramatically simplified laser-frequency stabilization architecture by direct feedback locking the laser to a reference gas cell without any external light control or modulation. This approach enables us to achieve a long-term laser frequency stability of ±6.5 MHz over 60 min.

## Results

### Laser design and basic performance

The demonstrated laser is based on hybrid integration between an InP reflective semiconductor optical amplifier (RSOA) gain chip and a thin-film lithium niobate (TFLN) photonic integrated circuit (PIC) that functions as an external cavity of the laser (Fig. 1a). TFLN PICs have recently attracted significant interest for broad applications^24–26^. Leveraging their electro-optic effect and quadratic optical nonlinearity for laser operation results in a novel type of integrated laser, namely integrated Pockels laser^27^, which opens up great potential for broad applications in a wide variety of photonic functionalities (Fig. 1a). However, integrated lasers previously demonstrated on the TFLN platform^27–37^ suffer from a fairly large laser linewidth and limited frequency chirping range, making them unsuitable for optical metrology applications.

To address this challenge, we employ the extended distributed Bragg reflector (eDBR) approach^38^ for single-mode lasing. In particular, we develop a novel type of eDBR structure (Fig. 1e, f) in which the Bragg grating is defined in the low-index silicon oxide cladding layer rather than in the TFLN layer itself. This novel approach allows flexible engineering of the Bragg scattering strength by simply controlling the layer thickness of the silicon oxide cladding and/or the etching depth of the Bragg grating (Fig. 1e, h). As silicon oxide has a refractive index considerably lower than TFLN while its plasma etching quality is significantly higher, this approach allows us to realize ultra-low Bragg scattering strength down to *κ* = 5.4 cm^−1^ over a long grating length of 1 cm, resulting in an ultra-narrow reflection spectrum as shown in Fig. 1h, ideal for narrow-linewidth lasing. For high-speed tuning of the laser frequency, a pair of tuning electrodes are integrated with the eDBR to tune its center wavelength, utilizing the electro-optic Pockels effect (*r*_33_) of TFLN. For MHF tuning the laser frequency, the driving electrodes are extended to a section of the TFLN waveguide, which effectively functions as a phase shifter to assist in the tuning of the resonance mode of the entire laser cavity (Fig. 1a). This co-tuned approach simplifies the electrical driving structure and laser operation. Compared with ring-resonator-based laser structures, the eDBR Pockels laser design mitigates the mode-mismatching issues caused by fabrication errors and enables plug-and-play functionality, facilitating more versatile laser tuning. As a result, its potential functionalities are significantly expanded, surpassing those of conventional laser approaches, as we demonstrate below. Details of the laser structure design are provided in the Method.

**Fig. 2:**
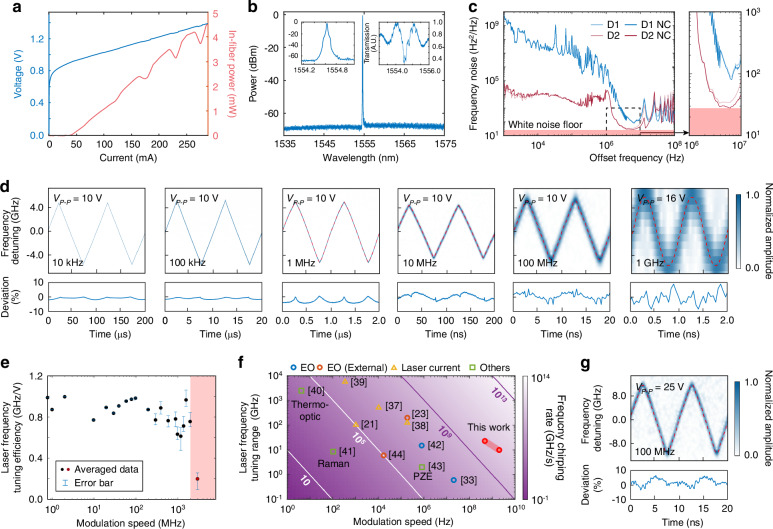
Characterization of laser performance. **a** Recorded light-current-voltage (LIV) curve of the laser, showing the fiber-coupled output power. The corresponding laser output power on chip is 5 dB higher, determined by the chip-to-fiber coupling efficiency. **b** Optical spectrum of single-mode lasing. The left inset shows a zoomed-in view of the spectrum. The right inset shows the measured transmission of the grating. **c** Recorded frequency noise spectra of two laser devices (D1, D2), with the white noise floor highlighted in red shading. NC: photodetector noise cancellation achieved via the cross-correlation technique. Right: zoomed-in spectra with the offset frequency from 1 to 10 MHz. **d** Recorded time–frequency spectrograms of the beat note between the Pockels laser and a reference diode laser at different modulation frequencies. The lower panels display the corresponding linearity deviation of the laser frequency chirping. The red dashed line in the figures represents the temporal waveform of the driving electrical signal, generated by an arbitrary waveform generator (AWG). For modulation speed ≥1 MHz, broadband electric amplifiers are used to boost the driving signal before it is applied to the laser. Limited amplifier bandwidths lead to certain waveform distortion on the driving electrical signal, particularly at high modulation frequencies. *V*_p−p_ shown on the figure indicates the peak-to-peak voltage of the driving electrical signal. Additional details are in Supplementary Information. **e** Recorded laser-frequency tuning efficiency versus modulation speed (i.e., modulation frequency). The red-shaded region indicates the frequency region where the tuning efficiency degrades. The error bars reflect measurement uncertainty from short-time Fourier transform processing due to instrument limitations. **f** Comparison of state-of-the-art laser frequency tuning performance. Red dots indicate results from our devices. **g** Similar to **d**, at a modulation speed of 100 MHz but with *V*_p−p_ of 25 V

With this approach, we achieve single-mode lasing operation with very narrow lasing linewidth. Figure 2 shows the laser performance. The laser exhibits a threshold current of 45 mA and emits an optical power of ~13.0 mW on chip (corresponding to 4.1 mW recorded in fiber) at a driving current of 260 mA (Fig. 2a). The laser operates around a telecom wavelength of 1555 nm, with a side-mode suppression ratio >58 dB (Fig. 2b). In particular, the laser exhibits an exceptionally narrow linewidth, as shown in Fig. 2c, with a white frequency noise floor down to 26.7 Hz^2^/Hz which corresponds to an intrinsic linewidth of only 167 Hz, a record value demonstrated on the TFLN platform^27–35^. Further details and a discussion of the device linewidth are included in the “Methods” section and Supplementary Information (SI).

High-speed frequency modulation of a laser is essential for a variety of optical metrology applications. To demonstrate this capability, we applied an electrical signal with a triangular waveform to drive the TFLN external laser cavity and monitored the laser frequency tuning by heterodyning the laser output with a stable narrow-linewidth continuous-wave reference laser. Figure 2d shows the recorded waveforms of the beating frequency at different modulation frequencies. It clearly demonstrates that the laser frequency tuning faithfully follows the driving electrical signal at all modulation frequencies up to 1 GHz. The blurring of the recorded spectrogram at high modulation speeds above 100 MHz is simply due to the limited sampling rate of the real-time oscilloscope used for recording the beating signal. The frequency chirping nonlinearity is <1% (root-mean-square (RMS) value) at modulation frequencies of 10 and 100 kHz. It slightly increases to 2.2–3.8% at higher modulation frequencies, probably caused by the electric amplifier used to boost the driving electrical signal (see the caption of Fig. 2d). An MHF tuning range of up to 10 GHz is achieved at all these modulation frequencies. The frequency tuning efficiency remains ~0.8 GHz/V for modulation speed up to 2 GHz (Fig. 2e) after which it begins to drop The degradation of tuning efficiency at higher modulation frequencies is likely related to the relative propagation delay between the laser wave and the driving electric signal inside the laser cavity, but its exact nature requires further exploration in the future. The frequency tuning range of 10 GHz at a modulation speed of 1 GHz corresponds to a frequency chirping rate as high as 2 × 10^19^ Hz/s. Figure 2f compares the frequency-tuning performance of the laser with state-of-the-art systems^21,23,33,39–46^. It clearly demonstrates that the recorded performance achieved here is the highest among existing lasers in terms of both frequency chirping rate and modulation speed. Interestingly, the MHF frequency tuning range increases considerably to 24 GHz at a modulation speed of around 100 MHz (Fig. 2g), which is more than one order of magnitude larger than previously reported values^27,33,34^. This improvement is likely due to the enhanced coordinated electro-optic tuning between the phase shifter and the eDBR around this frequency region (see SI for a detailed discussion of coordinated tuning).

### Distance metrology and velocimetry

The superior linear frequency tuning performance of the laser enables FMCW LiDAR with not only high-resolution ranging but also, particularly, velocimetry with a very large dynamic range. FMCW LiDAR relies on detecting the beating frequency between the reflected and reference laser waves to retrieve the distance (*R*) and velocity (*v*) information. The two beating frequencies during the laser frequency ramp-up and ramp-down duration are given by^4,5^

1 $${f}_{\uparrow }=\gamma \frac{2R}{c}-{\nu }_{{\rm {o}}}\frac{2v}{c}\equiv {f}_{\!{\rm {r}}}-{f}_{\!{\rm {d}}}$$

2 $${f}_{\downarrow }=\gamma \frac{2R}{c}+{\nu }_{{\rm {o}}}\frac{2v}{c}\equiv {f}_{\!{\rm {r}}}+{f}_{\!{\rm {d}}}$$

where *γ* is the frequency chirping rate, *ν*_o_ is the center laser frequency, and *c* is the velocity of light in vacuum. The first term ${f}_{{\rm {r}}}\equiv \gamma \frac{2R}{c}$ is the frequency difference introduced by the time delay due to light propagation, and the second term ${f}_{{\rm {d}}}\equiv {\nu }_{{\rm {o}}}\frac{2v}{c}$ represents the frequency shift induced by the Doppler effect. The detected distance and velocity are thus given by

3 $$R=\frac{c}{4\gamma }({f}_{\uparrow }+{f}_{\downarrow })\qquad v=\frac{c}{4{\nu }_{{\rm {o}}}}({f}_{\downarrow }-{f}_{\uparrow })$$

**Fig. 3:**
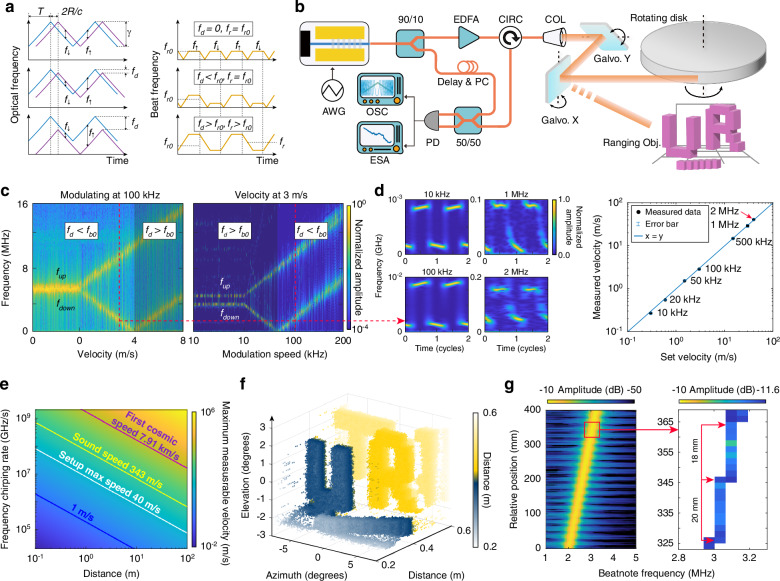
Ranging and velocimetry performance. **a** Schematic illustration of the time-dependent optical (left) and beatnote (right) frequency in an FMCW LiDAR. Top, middle, and bottom panels show three operational regimes with *f*_d_ = 0, *f*_d_ < *f*_r_, and *f*_d_ > *f*_r_, respectively. **b** Experimental setup for distance and velocity measurement. Photos of the objects are provided in SI. EDFA erbium-doped fiber amplifier, CIRC circulator, COL collimator, Galvo. Galvo mirror, PC polarization controller, PD photodetector, OSC oscilloscope, ESA electrical spectrum analyzer. **c** Left: Spectrum of the measured beat-not signal as a function of target velocity, with a fixed laser frequency modulation speed of 100 kHz. Right: As a function of laser frequency modulation speed, at a fixed target velocity of 3 m/s. The shaded areas indicate the false-detection regime due to insufficient laser frequency chirping rate. The red dashed line marks the case with a modulation speed of 100 kHz and a target velocity of 3 m/s, whose detailed spectrogram is shown in (**d**). **d** Time–frequency spectrogram of the beating signal at four different target velocities of 0.3, 3, 30, and 40 m/s. To showcase the effect of laser frequency modulation speed, the four spectrograms are measured with different laser-frequency modulation speeds of 10, 100 kHz, 1, and 2 MHz, respectively. The right figure compares the measured velocity by the device with the set velocity of the object. **e** Theoretical maximum measurable velocity for our laser. **f** 2D scanned ranging image of a static object. **g** Spectrum of the beatnote as a function of the relative position of an object, with a step of 2 mm. The red area is highlighted at right to show the ranging resolution

Equations (1)–(3) show that meaningful measurements of distance and velocity require both *f*_↑_ > 0 and *f*_↓_ > 0, which in turn requires ∣*f*_d_∣ < ∣*f*_r_∣, leading to $| v| < \frac{\gamma R}{{\nu }_{{\rm {o}}}}$. The detectable velocity is fundamentally limited by the laser frequency chirping rate and the ranging distance. If ∣*f*_d_∣ > ∣*f*_r_∣, FMCW LiDAR will produce incorrect distance and velocity values. Figure 3a illustrates this effect. In practice, *f*_↑_ and *f*_↓_ are required to be >~1 kHz to reduce the impact of the 1/*f*-noise of the optical detector, which further limits the dynamic range of velocimetry. In general, it is challenging for a conventional FMCW LiDAR to detect a fast-moving object at a short distance.

This challenge is elegantly resolved by our laser. To demonstrate this feature, we used the setup shown in Fig. 3b to perform velocimetry on a high-speed target: an 8-inch foam disk mounted on a high-speed DC motor with tunable rotation speed (see SI for a photo). The laser beam is aligned with the edge of the disk to capture the high tangential velocity. The laser frequency is modulated with a triangular waveform with a frequency chirping range of 10 GHz, but at different modulation speeds to test the ranging and velocimetry performance.

First, we fix the modulation speed at 100 kHz (which corresponds to *γ* = 2 PHz/s) and vary the target velocity between 0 and 8 m/s. Figure 3c (left) shows the recorded spectra of the beating signal. When *v* = 0, *f*_↑_ = *f*_↓_ = 5.31 MHz indicating an effective target distance of 0.4 m. As the target velocity increases, *f*_↓_ increases linearly with *v* while *f*_↑_ decreases linearly, as expected, until *v* = 4 m/s at which point *f*_↑_ approaches zero. With further increased *v*, both *f*_↑_ and *f*_↓_ increase in *v* since in this region, the Doppler frequency shift dominates over the time-delay-induced frequency difference, ∣*f*_d_∣ > ∣*f*_r_∣. Consequently, the LiDAR infers an incorrect distance longer than the real value and an incorrect velocity smaller than the real value (see Eq. (3)). This effect can be seen more clearly by fixing the target velocity at *v* = 3 m/s while varying the laser-frequency modulation speed from 10 to 200 kHz (corresponding to *γ* = 0.2−4 PHz/s), as shown in Fig. 3c (right). When the modulation speed is low, *f*_↑_ erroneously decreases with increasing modulation speed till approaching zero, after which it starts to increase linearly. This again demonstrates that the LiDAR will produce incorrect ranging and velocimetry information with an insufficient rate of laser frequency chirping.

To show the capability of detecting large velocities, we set the target velocity to ~40 m/s. Figure 3d shows the spectrograms of the recorded beating signal at different target velocities. These spectrograms clearly indicate that both *f*_↑_ and *f*_↓_ can be well resolved across all velocities with an adequate laser frequency chirping rate. The waveform distortion of the time-dependent beatnote frequency arises primarily from the nonlinearity of laser frequency chirping, which is, in turn, dominated by the waveform distortion of the driving electrical signal (see Fig. 2d and caption). This waveform distortion is generally the dominant factor in determining the precision of velocity measurements. For example, at a target velocity of 40 m/s, the recorded *f*_d_ = (*f*_↓_−*f*_↑_)/2 = 52.7 ± 2.0 MHz, which corresponds to a measured velocity of 41.0 ± 1.5 m/s. Overall, the measured velocities agree well with the set values throughout the velocity range of 0.3−40 m/s. 40 m/s corresponds to 89.5 miles/h, which covers almost the full range of vehicle velocities on the road, making this suitable for LiDAR applications in self-driving technology. Note that although we use different frequency chirping rates in Fig. 3d to showcase the chirping rates required for detecting different velocities, in practice, *γ* can be fixed at a sufficiently high value for the desired dynamic range of velocity measurements.

Since 40 m/s is the maximum velocity offered by the rotating disk, we cannot measure larger velocity values in the experiment. However, the high laser-frequency chirping rate implies that significantly higher velocities could be measured using this laser system. Figure 3e shows the expected measurable velocity of our laser at varying chirp rates and ranges. The velocity up to the first cosmic speed of 7.91 km/s can be measured by our laser at a distance of 1 m away, and the velocity up to ~1000 km/s could be measured for an object about 100 m away. This clearly shows the powerfulness of the demonstrated laser for velocimetry with an unprecedented dynamic range.

In addition to the velocimetry capabilities demonstrated above, the laser also offers high-ranging resolution. To illustrate this, the collimated laser beam is directed by the galvanometer (Galvo) mirrors onto a separate path to scan across a different object. The static object consists of two letters and a background board, spaced 8 cm apart, along with a series of blocks featuring 1 cm depth increments at the bottom to demonstrate depth resolution (see Fig. 3b. A photo of the object is provided in SI). The laser frequency modulation speed is set to 60 kHz to simplify data acquisition. Figure 3f shows the recorded two-dimensional image of the ranging object, revealing its detailed structures. Detailed distance calibration (Fig. 3g) shows a ranging resolution of <2 cm, consistent with the laser frequency chirp range of 10 GHz, which corresponds to a theoretical ranging resolution of 1.5 cm.

### Laser frequency stabilization

**Fig. 4:**
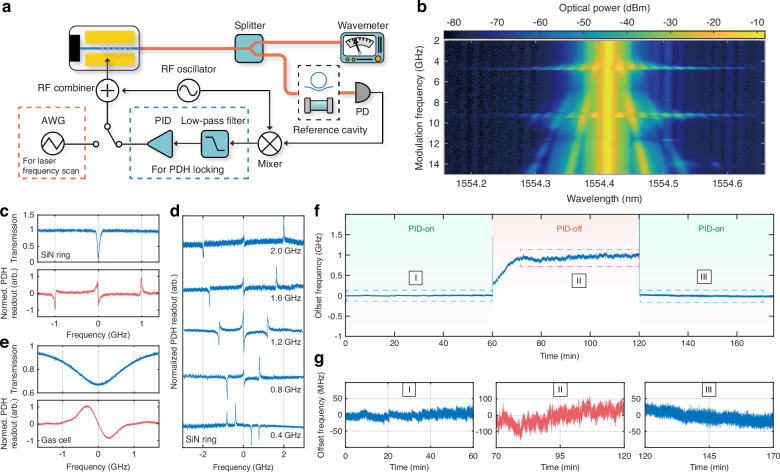
Laser frequency stabilization. **a** Schematic of the experimental setup for PDH laser locking. The reference cavity is either a high-*Q* silicon nitride microring resonator or a H^13^C^14^N gas cell. To obtain the laser-scanned transmission spectrum of the reference cavity (**c**, **e** Top figures), a triangular waveform signal, produced by an AWG at a modulation speed of 200 Hz, is used to drive the TFLN laser external cavity, so as to linearly scan the laser frequency at a slow speed. To obtain the laser-scanned PDH error signal (**c**, **e** Bottom figures and **d**), A single-frequency sinusoidal RF signal is added to drive the laser external cavity, together with the triangular waveform signal. For feedback locking the laser, the AWG is switched off and the PDH error signal is directed to a PID servo whose output is used to drive the laser's external cavity. **b** Optical spectrum of laser output when laser external cavity is modulated by a sinusoidal RF signal at different frequencies, with an RF power of 7 dBm. **c** Laser-scanned transmission spectrum (top) and PDH error signal (bottom) of a resonance mode of the reference silicon nitride microresonator. **d** Laser-scanned PDH error signal of the silicon nitride resonator, at different RF modulation frequencies. **e** Laser-scanned absorption spectrum (top) and PDH error signal (bottom) of the H^13^C^14^N P16 line of the reference gas cell. A RF modulation speed of 630 MHz is used to obtain the figure of the PDH error signal. **f** Time-dependent laser frequency, measured by a wavemeter (Toptica Photonics, WS6-600 VIS/IR-I). The PID servo is switched on in the first and third hour and is switched off in the second hour. **g** Detailed time-dependent laser frequency at the three time sections in dashed area in (**f**)

In addition to fast linear frequency chirping, the Pockels laser also supports high-speed phase modulation directly within the laser cavity. This capability is demonstrated in Fig. 4b, where a sinusoidal radio frequency (RF) driving signal is applied to the external TFLN laser cavity, with modulation speed varying between 2 and 15 GHz. Modulation sidebands are clearly visible on the optical spectrum of the laser output. At specific modulation speeds near 4.7 and 9.4 GHz which match with the free spectral range of the laser cavity, the RF modulation excites active fundamental and harmonic mode-locking, significantly broadening the optical spectrum. Beyond this, the sidebands evolve smoothly with increasing modulation speed across the full range of up to 15 GHz. To verify the nature of laser modulation, we remove the RSOA gain chip and launch a continuous-wave laser to the passive TFLN external cavity device, monitoring the transmission spectrum under identical modulation conditions (see SI). The close agreement between the spectra clearly verifies the nature of direct phase modulation within the laser cavity.

The high-speed phase modulation feature of the laser opens up a promising avenue for laser frequency stabilization with dramatically simplified architecture, as the embedded phase shifter eliminates the need for additional bulky external modulators and other alignment-sensitive components. To demonstrate this feature, we first use a high-*Q* silicon nitride microring resonator (intrinsic optical *Q* of 9 million) as a reference cavity and use the PDH technique to monitor the laser’s modulated output. To do so, we applied a triangular waveform driving signal with a low modulation speed of 200 Hz to scan the laser frequency. At the same time, we applied a sinusoidal RF signal to create modulation sidebands. Figure 4d shows the recorded PDH error signal at RF modulation frequencies ranging from 0.4 to 2.0 GHz, which exhibits a clear feature of the PDH error signal.

The clean PDH error signal directly produced by the laser indicates the great potential for direct PDH locking of the laser frequency. To confirm this, we switch the reference cavity to a fiber-coupled H^13^C^14^N gas cell. The laser wavelength is adjusted to be around the P16 absorption line near 1554.59 nm. Figure 3e shows the laser-scanned spectra of the P16 absorption line of H^13^C^14^N and the corresponding PDH error signal. To lock the laser frequency, we remove the triangular waveform RF signal and direct the produced PDH error signal to a proportional–integral–derivative (PID) servo controller whose output is combined with the sinusoidal RF signal to directly drive the TFLN external laser cavity. We record the laser frequency with a wavemeter, which is shown in Fig. 3f.

To show the repetitive direct locking of the laser frequency, we activate the PID servo for 60 min (Duration I in Fig. 4f), and then deactivate it for another 60 min (Duration II in Fig. 4f) during which the laser is free running. After that, we reactivate the PID servo for another 60 min (Duration III in Fig. 4f). As shown in Duration III, the laser frequency is stabilized to a near-constant value when the servo is on with a fluctuation of only ±13.1 MHz (RMS value) (Section III in Fig. 4g). This fluctuation is limited by the resolution of the wavemeter (Toptica Photonics, WS6-600 VIS/IR-I) which is about 20 MHz, suggesting a smaller actual instability. Further characterization with a higher-resolution wavemeter will be required to confirm this.

A small laser frequency drift of about 42.4 MHz over 50 min is observed, attributed to the mechanical drift of the two stages holding the RSOA and TFLN chips (Fig. 1g). To verify this, we manually corrected the mechanical drift during Duration I, by adjusting the stages by 50 nm approximately every 10 min. As shown in section I of Fig. 4g, this correction fully stabilizes the laser frequency over 60 min reducing fluctuations to ±6.5 MHz (RMS value) comparable to that of Duration III.

When the PID servo is disabled (Duration II), the laser frequency starts to drift as the laser is free-running. The laser frequency drifts by about 600 MHz within 10 min and then settles to a relatively stable value after which the laser frequency drifts slowly with an amount of ~113 MHz over a time duration of 50 min. Also, the laser frequency fluctuations increase to ±44.5 MHz (RMS value) (Section II in Fig. 4g). These results unequivocally validate the effectiveness of the demonstrated approach for direct laser frequency locking.

## Discussion

The frequency stability of the current laser is primarily determined by the mechanical stability of the test station since the RSOA gain chip and the TFLN PIC chip are placed on separate stages (Fig. 1g). We expect it to be significantly improved by adopting an appropriate packaging approach^47^ in the future, which would also further enhance the performance of laser frequency stabilization. On the other hand, in the current laser, we use a single set of driving electrodes to simultaneously tune the phase shifter and the eDBR. Although this simplifies the laser operation, it restricts the MHF tuning range because achieving large frequency shifts requires independent, coordinated adjustments of the phase shifter and eDBR (see SI for details). We project that the MHF tuning range could be substantially expanded by implementing dedicated driving electrodes to independently control the phase shifter and eDBR. Moreover, the frequency tuning efficiency of the laser can be further improved by using a longer EO phase-shifter section or by optimizing optical mode confinement to improve the EO modulation efficiency.

In summary, we have demonstrated a chip-scale integrated Pockels laser in the telecom band that exhibits a narrow intrinsic linewidth down to 167 Hz, delivers an optical power of 13 mW on-chip, and supports a broad MHF tuning range of 24 GHz. In particular, the laser achieves an unprecedented frequency chirping rate of up to 20 EHz/s and an enormous modulation bandwidth exceeding 10 GHz—both surpassing existing lasers by orders of magnitude. Using this laser, we demonstrated successful velocimetry at 40 m/s over a short distance of 0.4 m, inaccessible to conventional FMCW LiDAR, and distance metrology with a ranging resolution below 2 cm. Furthermore, to our knowledge, this work presents the first demonstration of a dramatically simplified laser frequency stabilization architecture, achieved through direct locking of the laser to an external reference gas cell without additional external light control. We successfully enabled long-term laser stability with a frequency fluctuation of only ±6.5 MHz over 60 min.

The outstanding performance of the demonstrated laser is poised to have profound impacts on a wide range of optical metrology applications. For example, in optical clocks, atomic sensors, and optical quantum computing systems, significant advances have recently been made in the miniaturization of optical reference cavities^48,49^, atomic vapor cells^50^, and ion traps^19^. However, the development of laser frequency control remains relatively limited. To date, lasers in these applications function almost exclusively as light sources whose control relies on complex and bulky systems, as discussed in the Introduction. This poses a major challenge, limiting the size, weight, and power consumption, thus hindering their wide deployment in practical environments. Since the majority of optical metrology applications require frequency-stabilized lasers for proper operation, the simplified architecture demonstrated here could significantly enhance both the integration and performance of these systems.

As another example, photonic Doppler velocimetry^51^ plays a crucial role in shock wave detection and dynamic compression studies, which are indispensable for applications such as inertial confinement fusion and explosive detonation detection, among others. However, detecting ultrahigh velocities remains challenging because the resulting extreme Doppler frequency shifts impose a significant burden on the operational bandwidth of optical detectors. This challenge could potentially be addressed with our laser, whose ultrafast frequency chirping can generate a delay-induced frequency to offset the Doppler frequency shift (see Eqs. (1) and (2)). Besides optical metrology applications, the demonstrated Pockels laser combines elegantly high laser coherence with ultrafast frequency reconfigurability and superior multifunctional capabilities that we envision to be highly promising for applications including communication, sensing, optical, and microwave synthesis, among many others.

## Method

### Laser structure design

The extended DBR laser structure comprises a gain section, a passive section connecting gain and EO-tunable components, a phase shifter section, and a tunable eDBR section. As shown in the schematic from Fig. 1a, a hybrid integration approach is implemented for the laser: a single-angled facet (SAF) III/V gain chip operating in the C-band is edge-coupled to an external cavity (EC) made on a TFLN PIC chip. A 5-μm-wide horn coupler at the EC facet is optimized to reduce the mode mismatch between the III–V gain chip and the LN waveguide, minimizing the intracavity losses. An angle of 10° is designed for the waveguide input facet to reduce the back-reflection of light. Laser frequency control is achieved by positioning a pair of electrodes along a 3-mm-long eDBR section and a 6-mm-long TFLN waveguide, the latter of which functions effectively as an electro-optic (EO) phase shifter. The TFLN waveguide has a width of 2.1 μm. A 3.0 μm gap between the waveguide and the gold electrode is designed to optimize the EO tuning efficiency while keeping the propagation loss intact. *V*_π_ of the EO phase shifter is measured to be about 6 V using a separate device with an identical structure. Details on section lengths optimized for mode-hop-free tuning are provided in Supplementary Information.

### Device fabrication

The devices were fabricated on a 600-nm-thick x-cut single-crystalline LN thin film bonded to a 4.7-μm silicon dioxide layer on a silicon substrate (from NanoLN). Fabrication began with waveguide patterning: a layer of ZEP520A resist was spin-coated onto the sample and baked, followed by electron beam lithography (EBL) to define the waveguide structure. The sample was then subjected to dry etching with argon ion (Ar^+^) to form ridge waveguides with an etch depth of 300 nm. A subsequent cleaning process removed resist and redeposition residues, resulting in smooth waveguide sidewalls. To define the eDBR grating in the demonstrated device, a 500-nm-thick SiO_2_ cladding was deposited using ICP-PECVD. A second EBL step was used to pattern the DBR structure, followed by reactive ion etching (RIE) to etch 150 nm into the cladding, creating the grating trenches. For electrode fabrication, SiO_2_ was first etched down to the LN layer using an additional lithography and etching step. Gold electrodes (500 nm thick) were then deposited via electron-beam evaporation, followed by a PMMA lift-off process. The chip facets were cut and polished to ensure smooth coupling with the gain chip.

### Laser linewidth measurement

The intrinsic linewidth of the laser is characterized by a variation of the self-heterodyne measurement^52^. A splitter separates the light into two paths in a Mach–Zehnder interferometer (MZI), with a 17 m delay line in one arm and an AOM in another. The AOM is modulated at 300 MHz, creating isolation of low-frequency environment noise from the measurement. The light output from the MZI is then detected by a balanced photodetector (BPD), and its output is recorded by a real-time oscilloscope. The phase noise is extracted from the captured waveform via the Hilbert transform and converted into the frequency noise, allowing identification of the white noise floor and the corresponding intrinsic laser linewidth. Additionally, to suppress BPD-introduced noise, a second BPD was added to the setup, and the cross-correlation technique was applied. Figure 2c shows the derived frequency noise spectra for D1 and D2 with BPD noise cancellation applied. Compared to the results without cancellation, a clear noise reduction was observed at the offset frequency at offset frequencies >5 MHz, resulting in a 36-Hz reduction in the measured intrinsic linewidth for both devices. A more detailed analysis is given in the SI.

## Supplementary information

Supplementary Information for Pockels Laser Directly Driving Ultrafast Optical Metrology

## Data Availability

All data are available in the main text or in the “Methods” section.

## References

[1] Ludlow, A. D., Boyd, M. M., Ye, J., Peik, E. & Schmidt, P. O. Optical atomic clocks. *Rev. Mod. Phys.* **87**, 637–701 (2015).

[2] Bailes, M. et al. Gravitational-wave physics and astronomy in the 2020s and 2030s. *Nat. Rev. Phys.* **3**, 344–366 (2021).

[3] Bertone, G. & Tait, T. M. A new era in the search for dark matter. *Nature* **562**, 51–56 (2018). 30283104 10.1038/s41586-018-0542-z

[4] Behroozpour, B., Sandborn, P. A., Wu, M. C. & Boser, B. E. Lidar system architectures and circuits. *IEEE Commun. Mag.* **55**, 135–142 (2017).

[5] Li, Y. & Ibanez-Guzman, J. Lidar for autonomous driving: the principles, challenges, and trends for automotive lidar and perception systems. *IEEE Signal Process. Mag.* **37**, 50–61 (2020).

[6] Dahiya, R. S., Metta, G., Valle, M. & Sandini, G. Tactile sensing-from humans to humanoids. *IEEE Trans. Robot.* **26**, 1–20 (2009).

[7] Shimizu, Y. et al. An insight into optical metrology in manufacturing. *Meas. Sci. Technol.* **32**, 042003 (2021).

[8] Liang, W. et al. Ultralow noise miniature external cavity semiconductor laser. *Nat. Commun.* **6**, 7371 (2015). 26104321 10.1038/ncomms8371 PMC4491184

[9] Boller, K.-J. et al. Hybrid integrated semiconductor lasers with silicon nitride feedback circuits. In *Photonics* Vol. 7, 4 (Multidisciplinary Digital Publishing Institute, 2020).

[10] Xiang, C., Bowers, S. M., Bjorlin, A., Blum, R. & Bowers, J. E. Perspective on the future of silicon photonics and electronics. *Appl. Phys. Lett*. **118** (2021).

[11] Han, Y., Park, H., Bowers, J. & Lau, K. M. Recent advances in light sources on silicon. *Adv. Opt. Photonics* **14**, 404–454 (2022).

[12] Porter, C., Zeng, S., Zhao, X. & Zhu, L. Hybrid integrated chip-scale laser systems. *APL Photonics* **8** (2023).

[13] Drever, R. W. et al. Laser phase and frequency stabilization using an optical resonator. *Appl. Phys. B* **31**, 97–105 (1983).

[14] Black, E. D. An introduction to Pound–Drever–Hall laser frequency stabilization. *Am. J. Phys.* **69**, 79–87 (2001).

[15] Alnis, J., Matveev, A., Kolachevsky, N., Udem, T. & Hänsch, T. Subhertz linewidth diode lasers by stabilization to vibrationally and thermally compensated ultralow-expansion glass fabry-pérot cavities. *Phys. Rev. A—At. Mol. Opt. Phys.* **77**, 053809 (2008).

[16] Schmid, F., Weitenberg, J., Hänsch, T. W., Udem, T. & Ozawa, A. Simple phase noise measurement scheme for cavity-stabilized laser systems. *Opt. Lett.* **44**, 2709–2712 (2019).

[17] Fan, H. et al. Atom based rf electric field sensing. *J. Phys. B: At. Mol. Opt. Phys.* **48**, 202001 (2015).

[18] Legaie, R., Picken, C. J. & Pritchard, J. D. Sub-kilohertz excitation lasers for quantum information processing with Rydberg atoms. *JOSA B* **35**, 892–898 (2018).

[19] Bruzewicz, C. D., Chiaverini, J., McConnell, R. & Sage, J. M. Trapped-ion quantum computing: progress and challenges. *Appl. Phys. Rev.* **6** (2019).

[20] Morgado, M. & Whitlock, S. Quantum simulation and computing with Rydberg-interacting qubits. *AVS Quantum Sci.* **3** (2021).

[21] Satyan, N., Vasilyev, A., Rakuljic, G., Leyva, V. & Yariv, A. Precise control of broadband frequency chirps using optoelectronic feedback. *Opt. Express* **17**, 15991–15999 (2009). 19724598 10.1364/OE.17.015991

[22] Gao, S., O’Sullivan, M. & Hui, R. Complex-optical-field lidar system for range and vector velocity measurement. *Opt. Express* **20**, 25867–25875 (2012). 23187404 10.1364/OE.20.025867

[23] Lu, Z. et al. Broadband linearly chirped light source with narrow linewidth based on external modulation. *Opt. Lett.* **43**, 4144–4147 (2018). 30160737 10.1364/OL.43.004144

[24] Lin, J., Bo, F., Cheng, Y. & Xu, J. Advances in on-chip photonic devices based on lithium niobate on insulator. *Photonics Res.* **8**, 1910–1936 (2020).

[25] Zhu, D. et al. Integrated photonics on thin-film lithium niobate. *Adv. Opt. Photonics* **13**, 242–352 (2021).

[26] Boes, A. et al. Lithium niobate photonics: unlocking the electromagnetic spectrum. *Science* **379**, eabj4396 (2023). 36603073 10.1126/science.abj4396

[27] Li, M. et al. Integrated Pockels laser. *Nat. Commun.* **13**, 5344 (2022). 36097269 10.1038/s41467-022-33101-6 PMC9467990

[28] Op de Beeck, C. et al. III/V-on-lithium niobate amplifiers and lasers. *Optica* **8**, 1288–1289 (2021).

[29] Gao, R. et al. On-chip ultra-narrow-linewidth single-mode microlaser on lithium niobate on insulator. *Opt. Lett.* **46**, 3131–3134 (2021). 34197398 10.1364/OL.430015

[30] Shams-Ansari, A. et al. Electrically pumped laser transmitter integrated on thin-film lithium niobate. *Optica* **9**, 408–411 (2022).

[31] Han, Y. et al. Widely tunable O-band lithium niobite/III–V transmitter. *Opt. Express* **30**, 35478–35485 (2022). 36258498 10.1364/OE.471402

[32] Lin, J. et al. Electro-optic tuning of a single-frequency ultranarrow linewidth microdisk laser. *Adv. Photonics* **4**, 036001–036001 (2022).

[33] Snigirev, V. et al. Ultrafast tunable lasers using lithium niobate integrated photonics. *Nature* **615**, 411–417 (2023). 36922611 10.1038/s41586-023-05724-2 PMC10017507

[34] Li, Z. et al. High density lithium niobate photonic integrated circuits. *Nat. Commun.* **14**, 4856 (2023). 37563149 10.1038/s41467-023-40502-8 PMC10415301

[35] Luo, Q., Bo, F., Kong, Y., Zhang, G. & Xu, J. Advances in lithium niobate thin-film lasers and amplifiers: a review. *Adv. Photonics* **5**, 034002–034002 (2023).

[36] Wang, S. et al. High-performance integrated laser based on thin-film lithium niobate photonics for coherent ranging. *Laser Photonics Rev.* **18**, 2400224 (2024).

[37] Franken, C. A. et al. High-power and narrow-linewidth laser on thin-film lithium niobate enabled by photonic wire bonding. *APL Photonics* **10** (2025).

[38] Huang, D. et al. High-power sub-kHz linewidth lasers fully integrated on silicon. *Optica* **6**, 745–752 (2019).

[39] Vasilyev, A., Satyan, N., Rakuljic, G. & Yariv, A. Terahertz chirp generation using frequency stitched VCSELs for increased LIDAR resolution. In *CLEO: Science and Innovations*, CF3C-1 (Optica Publishing Group, 2012).

[40] Behroozpour, B. et al. Electronic-photonic integrated circuit for 3d microimaging. *IEEE J. Solid-State Circuits* **52**, 161–172 (2016).

[41] DiLazaro, T. & Nehmetallah, G. Large-volume, low-cost, high-precision FMCW tomography using stitched DFBS. *Opt. Express* **26**, 2891–2904 (2018). 29401823 10.1364/OE.26.002891

[42] Zheng, J. et al. High-precision silicon-integrated frequency-modulated continuous wave lidar calibrated using a microresonator. *ACS Photonics* **9**, 2783–2791 (2022).

[43] Li, M. et al. Microcavity Raman laser-based FMCW lidar with enhanced echo sensitivity. *ACS Photonics* **11**, 801–809 (2024).

[44] Zhang, C. et al. High-fidelity sub-petabit-per-second self-homodyne fronthaul using broadband electro-optic combs. *Nat. Commun.* **15**, 6621 (2024). 39103469 10.1038/s41467-024-51103-4 PMC11300759

[45] Lihachev, G. et al. Low-noise frequency-agile photonic integrated lasers for coherent ranging. *Nat. Commun.* **13**, 3522 (2022). 35725718 10.1038/s41467-022-30911-6 PMC9209488

[46] Wang, N. et al. Inverse synthetic aperture ladar demonstration: system structure, imaging processing, and experiment result. *Appl. Opt.* **57**, 230–236 (2018). 29328169 10.1364/AO.57.000230

[47] Shen, B. et al. Integrated turnkey soliton microcombs. *Nature* **582**, 365–369 (2020). 32555486 10.1038/s41586-020-2358-x

[48] Lee, H. et al. Spiral resonators for on-chip laser frequency stabilization. *Nat. Commun.* **4**, 2468 (2013). 24043134 10.1038/ncomms3468 PMC3778514

[49] Zhao, Q. et al. Integrated reference cavity with dual-mode optical thermometry for frequency correction. *Optica* **8**, 1481–1487 (2021).

[50] Kitching, J. Chip-scale atomic devices. *Appl. Phys. Rev.* **5** (2018).

[51] Dolan, D. Extreme measurements with photonic Doppler velocimetry (PDV). *Rev. Sci. Instrum.* **91** (2020). 10.1063/5.0004363 32486719

[52] Yuan, Z. et al. Correlated self-heterodyne method for ultra-low-noise laser linewidth measurements. *Opt. Express* **30**, 25147–25161 (2022). 36237052 10.1364/OE.458109

