# Supplementary material for: Pockels laser directly driving ultrafast optical metrology
Source: Light Sci Appl. 2025 May 30;14:209. doi: 10.1038/s41377-025-01872-4 (PMC12122812; doi:10.1038/s41377-025-01872-4)
Supplement: Supplementary file 1 — Supplementary Information for Pockels Laser Directly Driving Ultrafast Optical Metrology [file 41377_2025_1872_MOESM1_ESM.pdf]

Supplementary Information for
Pockels Laser Directly Driving Ultrafast Optical Metrology

Shixin Xue,[1,*] Mingxiao Li,[2,*] Raymond Lopez-rios,[3] Jingwei Ling,[1] Jeremy Staffa,[3] Zhengdong Gao,[1] Qili Hu,[1] Tian Qiu,[3] Lin Chang,[2] Heming Wang,[2] Chao Xiang,[2] John E. Bowers,[2,†] and Qiang Lin[1,3,†]

[1]*Department of Electrical and Computer Engineering, University of Rochester, Rochester, NY 14627*
[2]*Department of Electrical and Computer Engineering, University of California Santa Barbara, Santa Barbara, CA 93106*
[3]*Institute of Optics, University of Rochester, Rochester, NY 14627*

In this supplementary material, we provide detailed information on the analysis of mode-hop-free laser frequency tuning, linearity analysis of laser frequency tuning, analysis of laser linewidth, derivation of relaxation resonance frequency, characterization of laser active mode locking, characterization of phase modulation of the TFLN external cavity, and photos of the objects used in the LiDAR experiments.

* These two authors contributed equally.
† e-mail: jbowers@ucsb.edu; qiang.lin@rochester.edu

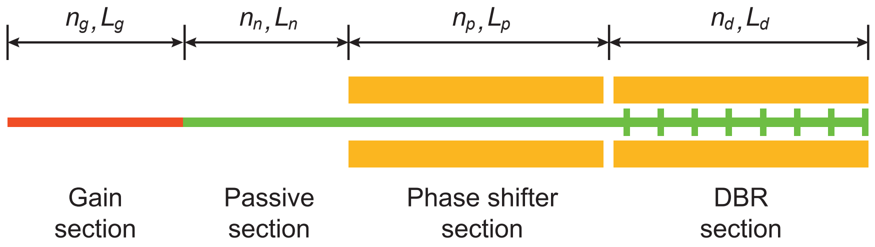

Fig. S1. **Schematic of EO-tunable eDBR external cavity laser.** The effective modal index and effective propagation length of each section are indicated in the figure.

I. DISCUSSION OF MODE-HOP-FREE TUNING WITH THE EO-TUNABLE EDBR EXTERNAL CAVITY LASER

This section introduces the requirement for mod-hop-free tuning in the eDBR Pockels laser. Fig. S1 illustrates conceptually the major waveguide sections of an eDBR laser. It consists of four major sections: a DBR section with a narrow reflection spectrum to confine the lasing wavelength, a phase shifter section to assist tuning the longitudinal mode of the whole laser cavity, a gain section formed by a semiconductor amplifier to provide optical gain, and a non-tuning passive section that connects the semiconductor amplifier with the TFLN external cavity. The resonance condition for the lasing wavelength λ_0 (wavelength in vacuum) is given by

$$n_g L_g + n_n L_n + n_p L_p + n_d L_d = \frac{m\lambda_0}{2} \tag{1}$$

where the effective length of the eDBR section is assumed to be L_d.

Both the eDBR and phase-shifter sections are tuned via the Pockels effect of TFLN, by applying an electric field across the LN waveguide to tune their modal refractive indices. On one hand, EO tuning the eDBR section will change the center wavelength λ_d of its reflection spectrum, given as

$$\frac{\delta\lambda_d}{\lambda_d} = \frac{\delta n_d}{n_d} \tag{2}$$

On the other hand, EO tuning the eDBR section and the phase shifter section will both alter the wavelength λ_l of the longitudinal mode of the whole laser cavity, which is given by

$$\frac{\delta\lambda_l}{\lambda_l} = \frac{\delta n_p L_p + \delta n_d L_d}{\bar{n}_g \bar{L}_g + n_p L_p + n_d L_d} \tag{3}$$

where we have simplified the notation of the whole non-EO-tuning section to be $\bar{n}_g \bar{L}_g \equiv n_g L_g + n_n L_n$.

The perfect mode-hop-free tuning of the lasing wavelength requires $\frac{\delta\lambda_l}{\lambda_l} = \frac{\delta\lambda_d}{\lambda_d}$. From Eqs. (2) and (3), this condition leads to

$$\frac{\delta n_p}{n_p} = \frac{\delta n_d}{n_d}\left(1 + \frac{\bar{n}_g \bar{L}_g}{n_p L_p}\right) \tag{4}$$

Equation (4) defines the requirement of coordinated tuning the eDBR section and the phase shifter section for mode-hop-free tuning the laser wavelength. In general, the phase shifter section and the DBR section are required to have different EO tuning efficiency to achieve a large mode-hop-free tuning of laser wavelength, determined by the ratio between the optical path length of the non-EO-tuning section and that of the phase shifter section.

In general, EO-induced refractive index change can be described as $\delta n_p = \eta_p \frac{V_p}{W_p}$ and $\delta n_d = \eta_d \frac{V_d}{W_d}$ where V_j $(j = p, d)$ is the driving voltage applied across the TFLN waveguide, W_j is the spacing between the driving electrodes, and η_j is the EO tuning rate which is determined by the specific EO waveguide structure. In our fabricated laser, the waveguide structures of the phase-shifter section and the DBR section are quite similar except that a shallow grating structure is defined in the SiO_2 cladding layer. As a result, $n_p \approx n_d$ and $\eta_p \approx \eta_d$. Equation (4) thus becomes

$$\frac{V_p}{V_d} \approx \frac{W_p}{W_d}\left(1 + \frac{\bar{n}_g \bar{L}_g}{n_p L_p}\right) \tag{5}$$

Equation (5) shows that there are two approaches to achieve large mode-hop-free laser tuning. On one hand, it can be realized using the same electrode spacing for the phase shifter and the DBR section, $W_p = W_d$, but applying different driving voltages with a ratio of $\frac{V_p}{V_d} \approx 1 + \frac{\bar{n}_g \bar{L}_g}{n_p L_p}$. On the other hand, it can be realized using different electrode spacings for the phase-shifter and DBR sections with a ratio of $\frac{W_d}{W_p} \approx 1 + \frac{\bar{n}_g \bar{L}_g}{n_p L_p}$, which will allow the use of a single driving voltage. This approach, however, will require precise electrode engineering, which will be explored in the future. In general, a long phase-shifter section with $n_p L_p \gg \bar{n}_g \bar{L}_g$ will help simplify the design and operation of mode-hop-free laser tuning.

II. CHARACTERIZATION OF LASER FREQUENCY TUNING LINEARITY

The linearity of the laser frequency tuning plays a critical role in ranging and velocimetry applications. Here, we further analyze the origins of nonlinearity in the laser frequency tuning. The time-frequency spectrograms shown in Fig. 2(d) of the main text are obtained using a short-time Fourier transform (STFT) of the recorded beat notes between the Pockels laser and the reference continuous-wave diode laser. We then retrieve the corresponding time-dependent beating frequency by extracting the frequency location of the beat note at each time instant from the spectrogram. These frequencies are plotted as blue curves in the top row of Fig. S2(a), where we also include the waveforms (red dashed curves) of the electrical signals used to drive the TFLN external laser cavity for direct comparison.

We first characterize the waveform distortion of the driving electrical signal, by comparing it with an ideal triangular waveform. The difference between the two, normalized by the peak-peak amplitude, is defined as Deviation 1 and plotted as red dashed curves in the middle row of Fig. S2(a) quantifying the magnitude of distortion in the driving signal. Deviation 1 serves as a reference for evaluating the linearity of laser frequency tuning later in the analysis. Fig. S2(a) clearly shows that the driving electrical signal closely approximates an ideal triangular waveform when the modulation frequency is 10 kHz and 100 kHz. This is because the electrical signal is directly produced by a high-performance arbitrary waveform generator (AWG), which produces high-fidelity triangular waveforms. However, the driving electrical signal deviates considerably from a triangular waveform when the modulation frequency becomes $\geq$1 MHz. This is primarily due to the limited bandwidth of the electrical amplifiers used to boost the signal amplitude, as the AWG does not provide adequate driving voltage amplitudes at high modulation frequencies.

Second, we perform the same process for the time-dependent beating frequency, by comparing it with a perfect triangular waveform. Their difference, normalized by the peak-peak amplitude, is defined as Deviation 2 (which is shown as blue curves in the middle row of Fig. S2(a)). The overall similarity between Deviation 2 (blue curves) and Deviation 1 (red dashed curves) indicates that the waveform distortion in the laser beating frequency stems primarily from that of the driving electrical signal.

The difference between Deviation 2 and Deviation 1 thus characterizes the linearity of laser frequency tuning. The results are plotted in the bottom row of Fig. S2(a). Note that these curves can be obtained directly by the waveform difference between the blue curves and the red dashed curves shown in the top row of Fig. S2(a) (after each is normalized by its own peak-peak amplitude). Therefore, they directly reflect the faithfulness of how the laser frequency tuning follows the driving electrical

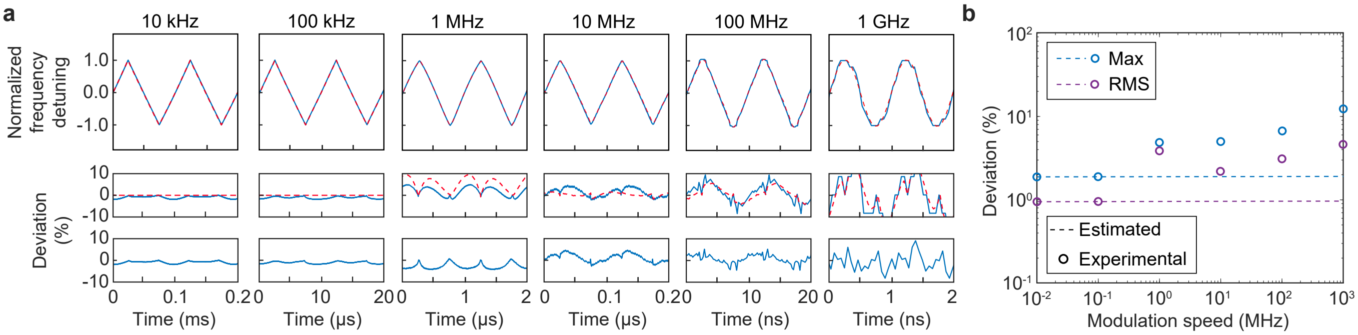

Fig. S2. **Characterization of the linearity of laser frequency tuning.** **(a)** Top row: Recorded waveform of the beat frequency (blue curve) and the corresponding driving electrical signal (red dashed curve). The blue curves are obtained by retrieving the frequency location of the time-frequency spectrograms (Fig. 2(d) of the main text) at each time instant. Middle row: Deviation of the driving electrical signal (normalized by its peak-to-peak amplitude) from an ideal triangular waveform, defined as Deviation 1 (red dashed curve). Deviation of the laser beat frequency (normalized by its peak-to-peak amplitude) from an ideal triangular waveform, which is defined as Deviation 2 (blue curve). Bottom row: Difference between Deviation 2 and Deviation 1. **(b)** Linearity deviation of laser frequency tuning, calculated from the curves shown in the bottom row of (a). The blue and purple dots represent the maximum value and the root-mean-square (RMS) value, respectively. The dashed lines indicate the values estimated from low modulation frequencies of 10 and 100 kHz, which represent the effect of non-coordinated phase-shifter and DBR tuning.

signal.

We used the curves in the bottom row of Fig. S2(a) to calculate the maximum amplitude and room mean square (RMS) value of each deviation curve, which are shown in Fig. S2(b). The laser frequency nonlinearity is fairly small at low modulation frequencies of 10 kHz and 100 kHz, with values of 1.97% for the maximum value and 0.98 % for the root-mean-square (RMS) value. This nonlinearity is primarily attributed to the lack of coordinated tuning between the laser longitudinal mode and the DBR grating mode, which, in combination with a certain laser frequency pulling effect, leads to a small nonlinearity in the laser frequency tuning. The nonlinearity increases when the modulation frequency increases to $\geq$1 MHz. It is likely introduced by the RF amplifiers employed, which may exhibit impedance mismatch with the RF driving electrode on the TFLN chip. As the TFLN external cavity device is capacitively driven (without termination), the impedance mismatch with the RF amplifier could result in certain residual RF signal that interferes with the source RF driving signal, leading to potential nonlinearity in laser frequency tuning. However, the exact underlying nature requires further exploration. When the modulation frequency increases to $>$1 GHz, the limited sampling rate of the real-time oscilloscope used to record the beating signal may affect the measurements, introducing additional apparent nonlinearity in the recorded data. Furthermore, at such a high modulation frequency, the relative propagation time delay of the laser waves and the driving electrical signal, within a round trip time of the laser cavity, could come into play. The precise impact of this delay also warrants further study.

III. ANALYSIS OF LASER LINEWIDTH

Narrow-linewidth high-coherence lasers are increasingly demanded in many applications including coherent communication, optical metrology, precision spectroscopy, and quantum computing[1–3]. As shown in main text Fig. 2(c), our Pockels lasers exhibit a linewidth at the hundreds-of-hertz level, enabled by the eDBR design. Here, we further analyze the linewidth difference between devices 1 and 2 in the main text Fig. 2(c).

The broadening of the laser linewidth is caused by the fluctuations in the phase of the optical field resulting from spontaneous emission and subsequent changes in the phase and intensity of lasing field. In addition to the instantaneous phase change directly related to the spontaneous emission, a delayed phase alter is caused by the change in field intensity at the same time. To restore the carrier-photon steady state, the laser will undergo a carrier density change, leading to a refractive index change and resulting in an additional phase shift of the laser field and line broadening. This process is described by the modified Schawlow-Townes-Henry equation as given below [4],

$$\Delta v = \frac{R_{sp}}{4\pi n_p} + \frac{R_{sp}}{4\pi n_p}\alpha_H^2 = \frac{R_{sp}}{4\pi n_p}(1+\alpha_H^2) \tag{6}$$

The first term originates from the direct phase fluctuation of the laser, while the second term is caused by the indirect intensity-introduced phase fluctuation. Moreover, the equation could be further modified as below to simplify the calculation for external cavity-based lasers [5],

$$\Delta v = \frac{\pi h v^3 n_{sp}}{P Q_L Q_E}(1+\alpha_H^2) \tag{7}$$

h is the Planck's constant, v is the lasing frequency, n_{sp} is the spontaneous emission factor, P is the emitted power, Q_E is the external quality factor of the laser cavity, Q_L is the loaded quality factor of the laser cavity, α_H is the linewidth enhancement factor. Q_L and Q_E are obtained from the equation below,

$$Q_E = \frac{2\pi n_g}{\alpha_m \lambda} \tag{8}$$

$$\alpha_m = \frac{-ln(R)}{L}, L = \frac{c}{n_g FSR} \tag{9}$$

$$Q_I = \frac{2\pi n_g}{\alpha_i \lambda} \tag{10}$$

$$\alpha_i = 2\alpha_{ec} + \alpha_{wg}\alpha_{ec} = \frac{-ln(l_{ec})}{L} \tag{11}$$

$$\frac{1}{Q_L} = \frac{1}{Q_I} + \frac{1}{Q_E} \tag{12}$$

λ is the lasing wavelength, n_g is the group velocity, L is the laser cavity length, R is the reflection ratio, FSR is the free spectral range of the laser, α_i (α_m) is the intrinsic (external/output coupling) loss of the laser, α_{ec} is the coupling loss between gain chip and eDBR chip, α_{wg} is the propagation loss in the laser cavity.

For D1 these values are $\lambda = 1.555$ μm, $P = 7.40$ mW, $n_g = 2.21$, $FSR = 4.2$ GHz, $\alpha_{wg} = 18.65$ dB/m, $R = 0.4$. For D2, we use the same setup and fabrication process with a similar design as for D1. The different values are the measured lasing wavelength $\lambda = 1.569$ μm, the emitted power $P = 7.75mW$ and the reflection ratio $R = 0.2$. Here, we assume the spontaneous emission factor $n_{sp} = 2.6$ [6]. Then the only unknown values are the coupling loss α_{ec} and linewidth enhancement factor α_H. To derive these two values, we sweep α_{ec} to calculate α_H using Eq. 7 with the measured intrinsic linewidth $\Delta\nu$ of 484 Hz for D1 and 167 Hz for D2 as given in the main text. It's important to note that the linewidth here is measured above the threshold power, hence the actual linewidth is half of the one derived from Schawlow-Townes-Henry equation [7]. The calculated results are shown in Fig. S3, where we obtain $\alpha_H = 4.04$ and $l_{ec} = 0.733$, corresponding to a coupling loss of 1.35 dB/facet, for the Pockels lasers demonstrated in this work.

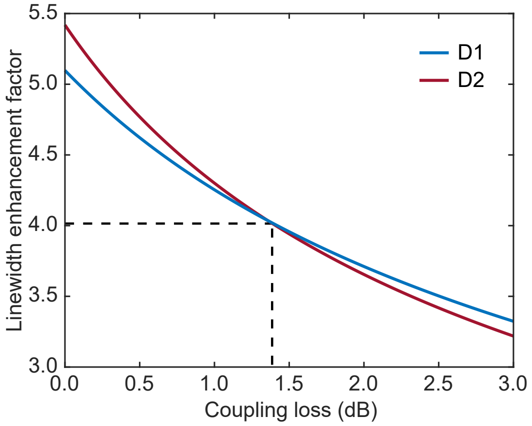

Fig. S3. **Analysis of laser linewidth enhancement factor.** Plot showing the linewidth enhancement factors of devices 1 and 2 as a function of the edge coupler loss, obtained by sweeping the loss parameter in the lasers.

IV. DISCUSSION OF VELOCIMETRY PERFORMANCE

Fig. S4(a) presents the time-frequency spectrograms of the recorded signal with laser frequency modulation speed up to 100 MHz. The waveform distortion of the time-dependent beatnote frequency is primarily introduced by the nonlinearity of laser

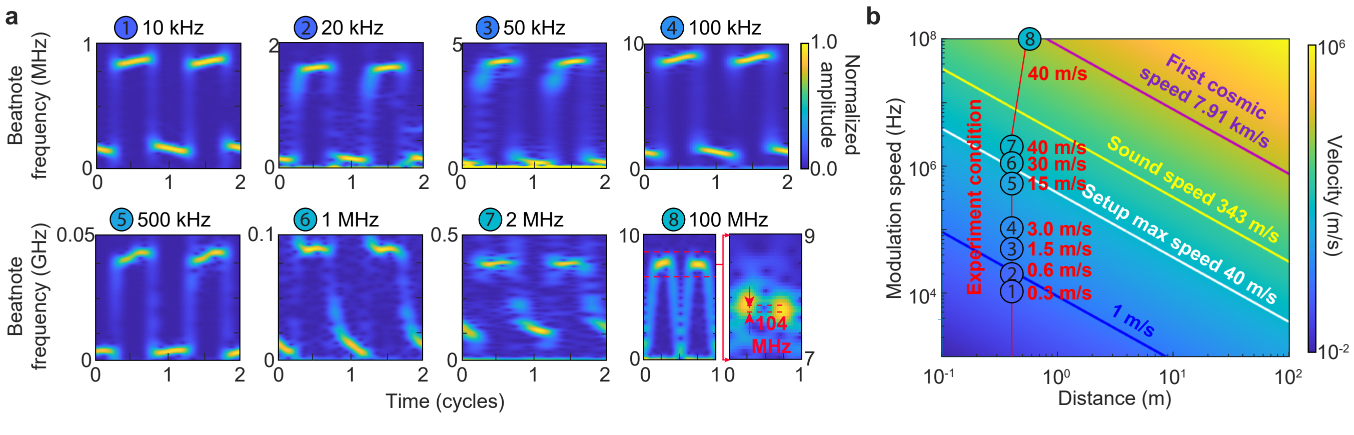

Fig. S4. **Characterization of velocimetry performance. (a)** Time-frequency spectrogram of the recorded signal, for set velocities ranging from 0.3 to 40 m/s. The employed speed for laser frequency modulation is indicated on the figures. For the 100 MHz case, the red dashed lines and the zoomed-in plots illustrate the detailed spectrogram. **(b)** Maximum measurable velocity with a laser frequency tuning range of 10 GHz. The red line highlights the experimental conditions corresponding to the measured set velocities. The background color of the tags indicates the speed of object according to the colormap.

frequency chirping which is in turn dominated by the waveform distortion of the driving electrical signal as shown clearly in Fig. S2. In general, such waveform distortion is the dominant factor determining the precision of velocity measurement.

In Fig. S4(a), the results with laser frequency modulation speed between 10 kHz and 2 MHz are used to plot the right figure of Fig. 3(d) in the main text. Since the experimental results match well with our calculations from Eq. (3) in main text, we further plot Fig. S4(b) to illustrate the theoretical expectation of the maximum measurable velocity, assuming a frequency tuning range of 10 GHz same as the experimental condition. The same figure is shown as Fig. 3(e) in the main text. Here, we align it with the experimental conditions for comparison. This figure clearly shows the great potential of the demonstrated laser for measuring extremely large velocities.

Note that, however, using a very high laser frequency chirping rate for velocimetry does come with a cost, since a high frequency chirping rate with a fixed frequency tuning range will lead to a short period of repetitive laser frequency modulation. It would limit the resolution of determining the beating frequencies $f_\uparrow$ and $f_\downarrow$ which in turn impacts the precision of velocity and distance measurement. This effect is illustrated in Fig. S4(a), by the case with a laser frequency modulation speed of 100 MHz for measuring the velocity of 40 m/s. The recorded $f_d = (f_\downarrow - f_\uparrow)/2 = 52 \pm 26$ MHz, which corresponds to a measured velocity of $v = 40.6 \pm 20.3$ m/s in which the measurement precision is now limited by the resolution in determining $f_\uparrow$ and $f_\downarrow$ as shown clearly in the spectrogram. This issue can be resolved by measuring $f_\uparrow$ and $f_\downarrow$ over multiple modulation periods instead of only one, a method widely employed in chirp-sequence FMCW RADAR [10, 11]. Its application for FMCW LiDAR will be developed in the next step in the near future.

V. CHARACTERIZATION OF ACTIVE MODE LOCKING OF THE LASER

Fig. S5 provides further detailed information about the active mode locking state of the laser when the frequency of the sinusoidal RF driving signal is around the free spectral range (FSR) of the laser cavity. Active mode locking of the laser significantly broadens the optical spectrum as shown in the main text and leads to strong pulsing in the time domain. Fig. S5(a)-(b) show coherent high-order harmonics in the RF spectrum of the detected laser output, indicating a clean periodic pulse train at the laser output. The active mode locking is also evident by the phase noise of the detected fundamental RF tone as shown in Fig. S5(c), which is very close to that of the RF signal used to drive the TFLN external cavity.

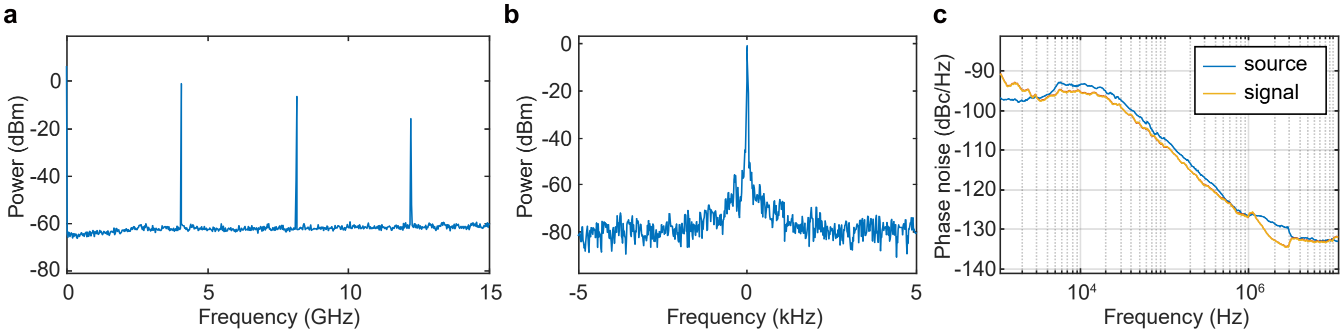

Fig. S5. **Characterization of active mode locking state of the laser.** **(a)** electrical spectrum of measured laser output when the TFLN external laser cavity is driven by a sinusoidal RF signal at a modulation frequency of 4.2 GHz. **(b)** Zoomed-in spectrum of the fundamental RF tone shown in (a). **(c)** Phase noise spectrum of the fundamental RF tone (red) and that of the RF driving source (blue).

VI. VERIFICATION OF DIRECT PHASE MODULATION INSIDE THE LASER CAVITY

To verify the nature of direct intracavity phase modulation of the eDBR laser as shown in the main text, we repeat the experiment on the passive TFLN external cavity device alone, as schematically shown in Fig. S6(a). The RSOA gain chip was replaced with a continuous-wave (CW) laser as the light source. A single-frequency sinusoidal RF signal is used to drive the TFLN external cavity device, same as what is done in the eDBR laser. The device output is monitored by an optical spectrum analyzer. As shown in Fig. S6(b), multiple orders of modulation sidebands are clearly visible on the spectrum, which evolve smoothly with increasing RF modulation frequency. This behavior closely resembles the eDBR laser's spectral evolution in Fig. 4(b) of the main text, except for the active mode locking dynamics near integer multiples of the cavity FSR. These results confirm that the spectral evolution shown in Fig. 4(b) of the main text arises from the intracavity phase modulation directly within the laser cavity.

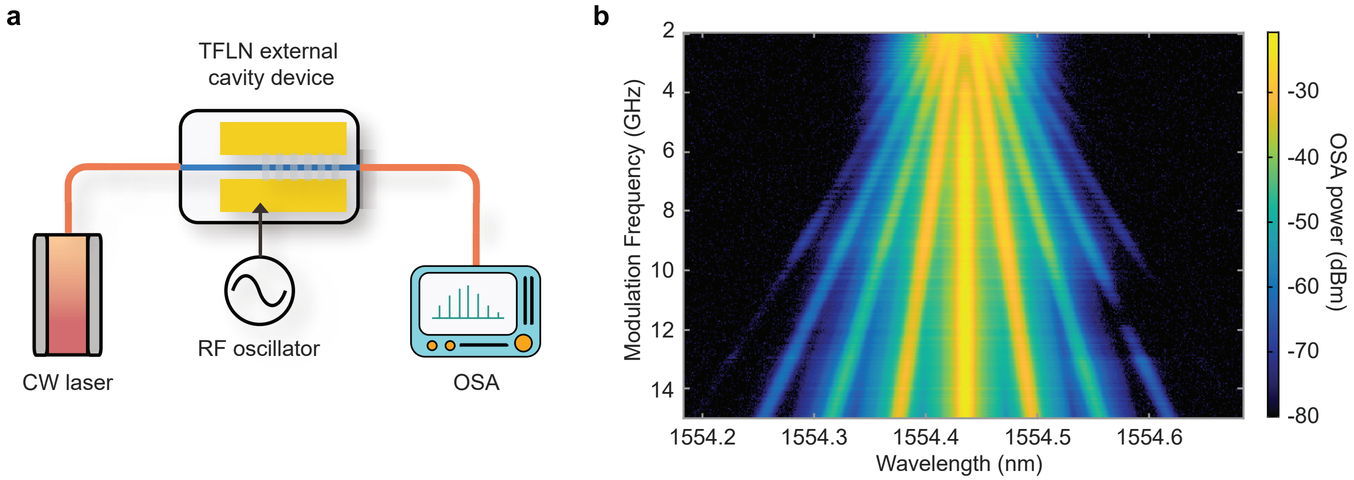

Fig. S6. **Phase modulation of the passive TFLN external cavity device.** **(a)** Schematic of the experimental setup, in which a continuous-wave (CW) laser serves as the light source and a single-frequency sinusoidal RF signal drives the passive TFLN external cavity device. **(b)** Optical spectrum of the device output at different RF modulation frequencies. The spectral bandwidth here is broader than that shown in Fig. 4(b) of the main text due to the use of a higher RF driving power (27 dBm).

VII. OBJECTS USED IN THE RANGING AND VELOCIMETRY EXPERIMENTS

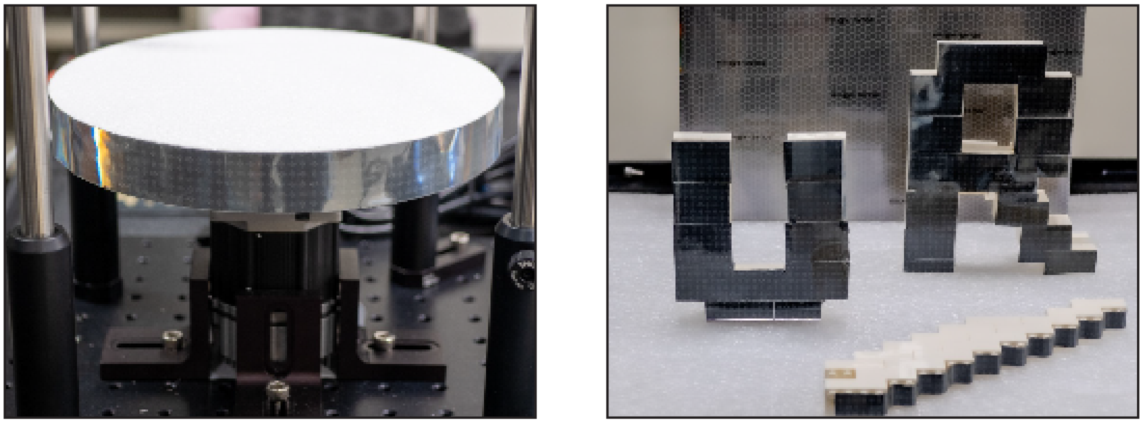

Fig. S7. **Objects used in the ranging and velocimtry experiments.** Left: Rotating foam disk used in the velocimetry experiment. Right: Object used for the 2D-scanned ranging experiment.

For the LiDAR velocimetry experiment, we use a foam disk with a diameter of 8 inch, mounted on a servo-motor (ClearPath-SCHP) to create adjustable tangential speed. The object is shown in the left of Fig. S7. For LiDAR ranging experiment, we use building blocks to create the “UR” letter and step objects. The object is shown in the right of Fig. S7. All targets are covered with retro tapes (Oralite V82) on the surface facing the incoming laser beam.

[1] D. T. Spencer, T. Drake, T. C. Briles, J. Stone, L. C. Sinclair, C. Fredrick, Q. Li, D. Westly, B. R. Ilic, A. Bluestone, N. Volet, T. Komljenovic, L. Chang, S. H. Lee, D. Y. Oh, M.-G. Suh, K. Y. Yang, M. H. P. Pfeiffer, T. J. Kippenberg, E. Norberg, L. Theogarajan, K. Vahala, N. R. Newbury, K. Srinivasan, J. E. Bowers, S. A. Diddams, S. B. Papp, An optical-frequency synthesizer using integrated photonics. *Nature* **557**, 81-85 (2018).

[2] D. Thomson, A. Zilkie, J. E. Bowers, T. Komljenovic, G. T. Reed, L. Vivien, D. Marris-Morini, E. Cassan, L. Virot, J.-M. Fédéli, J.-M. Hartmann, J. H. Schmid, D.-X. Xu, F. Boeuf, P. O’Brien, G. Z. Mashanovich, and M. Nedeljkovic, Roadmap on silicon photonics. *J. Opt.* **18**, 073003 (2016).

[3] E. Pelucchi, G. Fagas, I. Aharonovich, D. Englund, E. Figueroa, Q. Gong, H. Hannes, J. Liu, C.-Y. Lu, N. Matsuda, J.-W. Pan, F. Schreck, F. Sciarrino, C. Silberhorn, J. Wang, K. D. Jöns, The potential and global outlook of integrated photonics for quantum technologies. *Nat. Rev. Phys.* **4**, 194 (2022).

[4] L. Coldren, Diode Lasers and Photonic Integrated Circuits (3rd Ed.), *John Wiley & Sons*, (2012).

[5] M. A. Tran, D. Huang, and J. E. Bowers, *APL Photonics* **4**, 111101 (2019).

[6] C. H. Henry, Theory of linewidth of semiconductor lasers.*IEEE J. Quantum Electron.* **18**, 259 (1982).
[7] M. Lax, “Classical noise. V. Noise in self-sustained oscillators”, Phys. Rev. 160 (2), 290 (1967);
[8] D. Huang et al., ”Sub-kHz Linewidth Extended-DBR Lasers Heterogeneously Integrated on Silicon,” 2019 Optical Fiber Communications Conference and Exhibition (OFC), San Diego, CA, USA, 2019, pp. 1-3.
[9] Daryl T. Spencer, Mike Davenport, Sudharsanan Srinivasan, Jacob Khurgin, Paul A. Morton, and John E. Bowers, ”Low kappa, narrow bandwidth Si3N4 Bragg gratings,” Opt. Express 23, 30329-30336 (2015)
[10] G. Hakobyan and B. Yang, “High-Performance Automotive Radar,” IEEE Signal Processing Magazine **36**, 32 - 44 (2019).
[11] K. Doris, A. Filippi, and F. Jansen, “Reframing Fast-Chirp FMCW Transceivers for Future Automotive Radar: The pathway to higher resolution,” IEEE Solid-State Circuits Magazine **14**, 44 - 55 (2022).
